# Psf3 as a possible biomarker of postoperative chemotherapy for patients with early pulmonary adenocarcinoma

**DOI:** 10.1111/1759-7714.13230

**Published:** 2019-10-22

**Authors:** Kenji Kimura, Yugo Tanaka, Shunsuke Tauchi, Yoshitaka Kitamura, Wataru Nishio, Yasuhiro Sakai, Yoshitake Hayashi, Masahiro Yoshimura, Yoshimasa Maniwa

**Affiliations:** ^1^ Division of Thoracic Surgery Kobe University Graduate School of Medicine Kobe Japan; ^2^ Division of Thoracic Surgery Akashi Medical Center Akashi Japan; ^3^ Division of Thoracic Surgery Hyogo Cancer Center Akashi Japan; ^4^ Division of Pathology Hyogo Cancer Center Akashi Japan; ^5^ Division of Molecular and Medical Genetics, Department of Pathology Kobe University Graduate School of Medicine Kobe Japan

**Keywords:** Adjuvant chemotherapy, biomarker, lung cancer

## Abstract

**Background:**

Partner of Sld five 3 (Psf3) is a member of the heterotetrameric complex that consists of SLD5, Psf1, Psf2, and Psf3. We have shown in previous studies that high Psf3 expression was a poor prognostic marker for pulmonary adenocarcinoma. Here, we statistically evaluated the relationship between clinicopathologic factors and Psf3 expression in stage I pulmonary adenocarcinoma.

**Methods:**

A total of 583 patients who had undergone complete resection of stage I pulmonary adenocarcinoma from January 2002 to December 2009 were included in the study. Tissue microarrays were performed, and the resected tumors were divided into groups according to Psf3 expression.

**Results:**

Of 583 patients, high expression of Psf3 was observed in 211 (36.2%) and low expression of Psf3 observed in 372 (63.8%) patients. Among stage I patients, the five‐year survival rate was 76.7% in the Psf3 high expression group and 90.9% in the Psf3 low expression group (*P* < 0.0001). On multivariate analysis, Psf3 was found to be the independent prognostic factor. Among stage I patients in the Psf3 high expression group, a significantly greater five‐year survival rate was observed in patients who received postoperative chemotherapy with tegafur‐uracil than in those who underwent surgery alone (*P* < 0.0001). In contrast, among stage I patients in the Psf3 low expression group, no difference was found in the five‐year survival, regardless of the presence or absence of tegafur‐uracil (*P* = 0.873).

**Conclusion:**

The Psf3 expression was an independent prognostic factor and could be a biomarker of adjuvant tegafur‐uracil for stage I pulmonary adenocarcinoma.

**Key points:**

**Significant findings of the study:** The Psf3 expression could be a biomarker of adjuvant tegafur‐uracil administration for stage I pulmonary adenocarcinoma.

**What this study adds:** Appropriate patients of adjuvant chemotherapy for stage I pulmonary adenocarcinoma using Psf3 expression could be selected.

## Introduction

The opportunities for early detection of pulmonary cancer have been increasing due to the progress in diagnostic imaging, such as chest computed tomography (CT). Furthermore, a recent study demonstrated the efficacy of low‐dose CT screening for primary pulmonary cancer.^1^, ^2^, ^3^ Therefore, early‐stage pulmonary cancer is expected to have an increase in detection rates and would need further study in the future.

For early‐stage non‐small cell lung cancer (NSCLC), the most effective treatment has been surgery, with five‐year survival rates approximately 85% for stage IA and 75% for stage IB.^4^ Although the surgical outcome for early‐stage pulmonary cancer has been good, recurrence after surgery remains recognized at a certain rate. In Japan, tegafur‐uracil (UFT) had been used as postoperative therapy for stage I patients with tumor diameter of ≥2 cm.^5^, ^6^, ^7^ Although UFT can improve the survival of patients with stage I NSCLC, most patients may remain free of recurrence without the use of UFT. Therefore, we considered that selection of patients with poor prognosis at the time of surgery who may benefit from postoperative chemotherapy would improve the outcome for early‐stage pulmonary cancer. However, at the present time, there are no useful biomarkers that predict the prognosis of early pulmonary cancer.

Partner of Sld five 3 (Psf3) is one of the proteins known to comprise Go‐Ichi‐Ni‐San (GINS), which is a macromolecular protein complex associated with DNA replication. Although our previous studies have shown that Psf3 expression worked as a prognostic indicator in pulmonary adenocarcinoma,^8^, ^9^ its relationship with the efficacy of adjuvant chemotherapy was not determined.

In the present study, we retrospectively analyzed the value of Psf3 as a prognostic indicator in patients with early‐stage pulmonary cancer after surgery. Furthermore, we examined the association between Psf3 expression and the efficacy of adjuvant administration of UFT.

## Methods

### Patients

From January 2002 to December 2009, 583 consecutive patients with pathologic stage I pulmonary adenocarcinoma, for which complete resection was performed at the Hyogo Cancer Center, were included in the study. Tissue microarrays were obtained from all patients. Staging was based on the TNM classification of the seventh edition of the American Joint Committee on Cancer Staging Manual and the Revised International System for staging lung cancer. Patients who received preoperative chemotherapy and/or radiation therapy were excluded. Among patients who received postoperative therapy, 124 patients who received UFT (daily tegafur at 250 mg/m^2^ body surface area) were included, but those who received other platinum‐based chemotherapy and/or radiotherapy were excluded. Patients were followed‐up for a median period of 98 months. The protocol was implemented in accordance with the principles of the Declaration of Helsinki with the approval of the Clinical Research Area Ethics Committee of Kobe University Graduate School of Medicine (#180146) and the Hyogo Cancer Center (#R‐699). Clinical information was collected and prognostic factors were assessed retrospectively. Written informed consent was obtained from all patients.

### Tissue microarray and immunohistochemistry

Tissue microarrays and immunohistochemistry were performed as previously described.^9^ For the microarrays, tissue sections taken from the resected specimens that represented the largest cleaved surface of the tumor mass were cut into small circles. Plural circular sections were placed on a single slide, deparaffinized with xylene, and rehydrated with ethanol. For immunohistochemistry, the specimens were placed in Dako REALTarget Retrieval Solution (Dako, Glostrup, Denmark) at 98°C for 20 minutes for antigen retrieval.^8^ Mouse antihuman Psf3 monoclonal antibodies (1:500; GeneStem, Osaka, Japan) were used to detect Psf3. The Dako LSAB 2 Universal (DAB) kit (Dako) was used for endogenous peroxidase blocking, treatment with secondary antimouse and antirat immunoglobulin antibodies, and visualization of horseradish peroxidase. The samples were counterstained with hematoxylin. Photographs of the stained sections were obtained using a camera mounted on a Keyence BZ‐8000 digital microscope (Keyence, Osaka, Japan).

### Classification of immunohistochemical staining patterns

In this study, the Psf3 expression was determined using a microarray as described in previous studies.^9^ The cutoff value was 50% staining. If more than 50% of cancer cells in the microarray tissue samples showed nuclear staining and exhibited a clustering pattern of Psf3 in the microscope area (×200), the tissue was classified as high expression of Psf3; if not, the tissue was classified as low expression of Psf3. Two researchers (Y. S. and Y. H.), who had no knowledge of the patients' outcome evaluated all samples.

### Statistical analysis

Unless otherwise specified, the summarized data are presented as numbers or means ± standard deviation. Categorical and continuous variables were compared using the χ^2^ test and unpaired *t*‐test, respectively. Overall survival (OS) was defined as the time from surgery to death or the last follow‐up date. Disease‐free survival (DFS) was defined as the time from surgery to the detection of signs or symptoms of recurrence or the last follow‐up date. OS was evaluated by univariate and multivariate analyses. OS and DFS were calculated using the Kaplan‐Meier method and compared using the log‐rank test. Variables with *P*‐values <0.05 in the univariate analysis were subjected to multivariate analysis, using Cox regression models to calculate P values and hazard ratios. EZR (Saitama Medical Center, Jichi Medical University, Saitama, Japan) was used for statistical analyses of the data in this study.^10^

## Results

We examined the relationship between Psf3 expression and the clinicopathologic characteristics of 583 patients with primary pulmonary adenocarcinoma, pathologic stage I. Among the patients, 211 cases (36.2%) were high expression of Psf3 and 372 cases (63.8%) were low expression of Psf3. The proportions of patients with vascular, lymphatic vessel, and pleural invasion and heavy smoking were higher in the Psf3 high expression group than in the Psf3 low expression group (Table 1).

**Table 1:** Association between Psf3 expression and clinicopathologic characteristics in patients with stage I pulmonary adenocarcinoma (*N* = 583)

Variable	Total	Psf3		*P*‐value
		Low‐positive	High‐positive	
No. of patients	583	372	211	
Age (mean ± SD) (range)	66.3 ± 9.16 (34–84)	66.858 ± 8.94 (34–84)	65.346 ± 9.45 (38–84)	0.0556
Gender				
Male	309	170	139	<0.0001
Female	274	202	72	
Smoking status				
Pack years <40	412	284	128	<0.0001
Pack years ≥40	171	88	83	
Procedure				0.264
Lobectomy	410	253	157	
Segmentectomy	120	83	37	
Wedge resection	53	36	17	
T factor				0.00026
T1a	212	152	60	
T1b	186	123	63	
T2a	185	97	88	
Vessel invasion				<0.0001
Negative	456	324	132	
Positive	127	48	79	
Lymphatic invasion				<0.0001
Negative	476	329	147	
Positive	107	43	64	
Pleural invasion				<0.0001
Negative	488	329	159	
Positive	95	43	52	
Adjuvant administration of UFT				0.00886
Negative	456	304	152	
Positive	127	68	59	

Psf3, partner of Sld five 3; SD, standard deviation; UFT, tegafur‐uracil.

**Figure 1:**
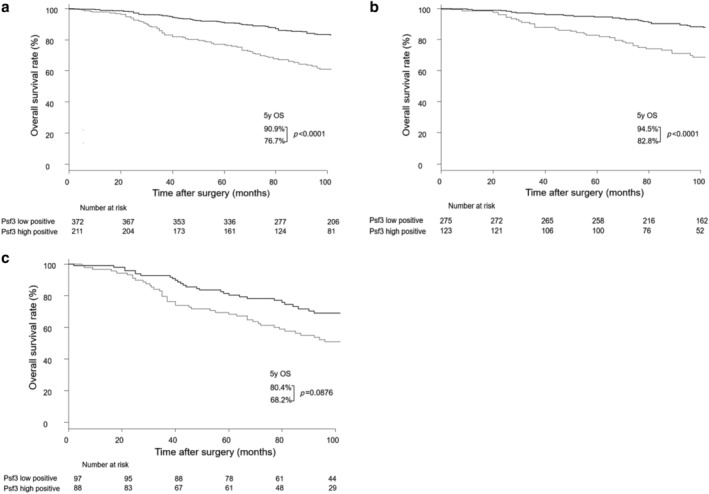
(**a**) Survival curve in patients with stage I pulmonary adenocarcinoma (*N* = 583), (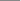) Psf3 low positive (*n* = 372) and (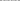) Psf3 high positive (*n* = 211), (**b**) Survival curve in patients with stage IA pulmonary adenocarcinoma according to Psf3 expression (*N* = 398), (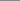) Psf3 low positive (*n* = 275) and (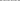) Psf3 high positive (*n* = 123), and (**c**) Survival curve in patients with stage IB pulmonary adenocarcinoma, according to Psf3 expression among stage IB patients (*N* = 185). (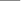) Psf3 low positive (*n* = 97) and (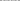) Psf3 high positive (*n* = 88).

Among the stage I patients, the five‐year OS rate was 76.7% in the Psf3 high expression group and 90.9% in the Psf3 low expression group (*P* < 0.0001; Fig 1a). We further analyzed the correlation of survival and Psf3 expression, according to more detailed staging. Among stage IA patients, a significantly worse five‐year survival rate was also found in the Psf3 high expression group than in the Psf3 low expression group (82.8% vs. 94.5%, *P* < 0.0001; Fig 1b). Among stage IB patients, there was a tendency of an association between OS and Psf3 expression, but significant differences were not observed between the two groups (68.2% vs. 80.4%, respectively; *P* = 0.0876; Fig 1c).

**Figure 2:**
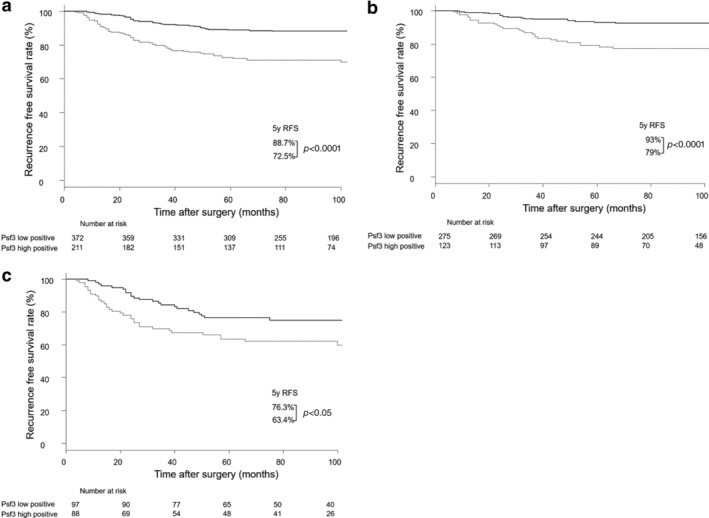
(**a**) Recurrence‐free survival curve in patients with stage I pulmonary adenocarcinoma (*N* = 583), (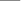) Psf3 low positive (*n* = 372) and (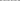) Psf3 high positive (*n* = 211), (**b**) Recurrence‐free survival curve in patients with stage IA pulmonary adenocarcinoma (*N* = 398), (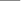) Psf3 low positive (*n* = 275) and (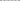) Psf3 high positive (*n* = 123), and (**c**) Recurrence‐free survival curve in patients with stage IB pulmonary adenocarcinoma, according to Psf3 expression among stage IB patients (*N* = 185). (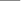) Psf3 low positive (*n* = 97) and (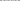) Psf3 high positive (*n* = 88).

Among stage I patients, the five‐year recurrence‐free survival (RFS) rate was significantly lower in the Psf3 high expression group than in the Psf3 low expression group (72.5% vs. 88.7%, *P* < 0.0001; Fig 2a). Likewise, the five‐year RFS was significantly worse in the Psf3 high expression group than in the Psf3 low expression group among patients in stage IA (79% vs. 93%, *P* < 0.0001; Fig 2b) and stage IB (63.4% vs. 76.3%, *P* < 0.05; Fig 2c). On univariate analysis, gender, vascular invasion, lymphatic vessel invasion, pleural invasion, Psf3 expression, and pathologic T factor were the poor prognostic factors (Table 2). On multivariate analysis, older age, male sex, and high expression of Psf3 were the significant independent predictors of worse outcomes (Table 3).

**Table 2:** Univariate analysis of the association between overall survival and prognostic factors in stage I pulmonary adenocarcinoma by the Cox proportional hazards model (*N* = 583)

Variable	HR	95% CI	*P*‐value
Gender (male vs. female)	2.013	1.43–1.910	<0.0001
Age (≥70 vs. <70 years)	2.577	1.867–3.560	<0.0001
Resection procedure (nonanatomical vs. anatomical)	1.585	0.991–2.536	≤0.05
T factor (T2 vs. T1)	2.501	1.826–3.425	<0.0001
Vessel invasion (positive vs. negative)	2.455	1.762–3.420	<0.0001
Lymphatic invasion (positive vs. negative)	2.189	1.554–3.084	<0.0001
Pleural invasion (positive vs. negative)	2.803	1.989–3.950	<0.0001
Psf3 (high‐positive vs. low‐positive)	2.373	1.732–3.252	<0.0001
Postoperative chemotherapy [UFT(−) vs. UFT(+)]	0.707	0.457–1.093	0.1186

Anatomical resection: segmentectomy, lobectomy, bilobectomy, or pneumonectomy; CI, confidence interval; HR, hazard ratio; nonanatomical resection: wedge resection; UFT, tegafur‐uracil.

**Table 3:** Multivariate analysis of the association between overall survival and prognostic factors of patients with stage I pulmonary adenocarcinoma by the Cox proportional hazards model (*N* = 583)

Variable	HR	95% CI	*P*‐value
Gender (male vs. female)	1.556	1.095–2.212	0.014
Age (≥70 vs. <70 years)	2.728	1.964–3.787	<0.0001
T factor (T1 vs. T2)	1.363	0.898–2.071	0.1463
Vessel invasion (positive vs. negative)	1.365	0.907–2.054	0.1353
Lymphatic invasion (positive vs. negative)	1.186	0.801–1.758	0.395
Pleural invasion (positive vs. negative)	1.576	0.984–2.524	0.0586
Psf3 (high‐positive vs. low‐positive)	1.872	1.324–2.645	<0.0001

CI, confidence interval; HR, hazard ratio.

**Figure 3:**
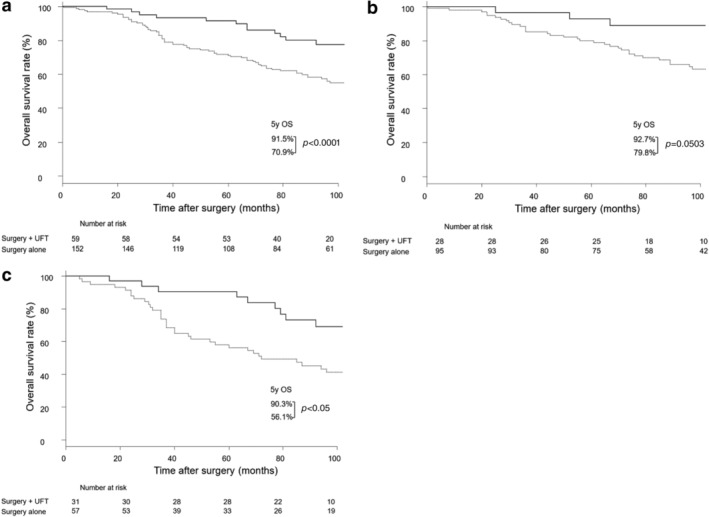
(**a**) Survival curve among patients with stage I pulmonary adenocarcinoma with high‐positive Psf3 expression and who received adjuvant UFT (*N* = 211). (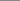) Surgery + UFT (*n* = 59) and (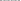) surgery alone (*n* = 152). (**b**) Survival curve among patients with stage IA pulmonary adenocarcinoma with high expression of Psf3 and who received adjuvant UFT (*N* = 123). (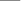) Surgery + UFT (*n* = 28) and (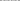) surgery alone (*n* = 95). (**c**) Survival curve among patients with stage IB pulmonary adenocarcinoma with high expression of Psf3 and who received adjuvant UFT (*N* = 88). (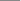) Surgery + UFT (*n* = 31) and (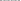) surgery alone (*n* = 57). UFT, tegafur‐uracil.

**Figure 4:**
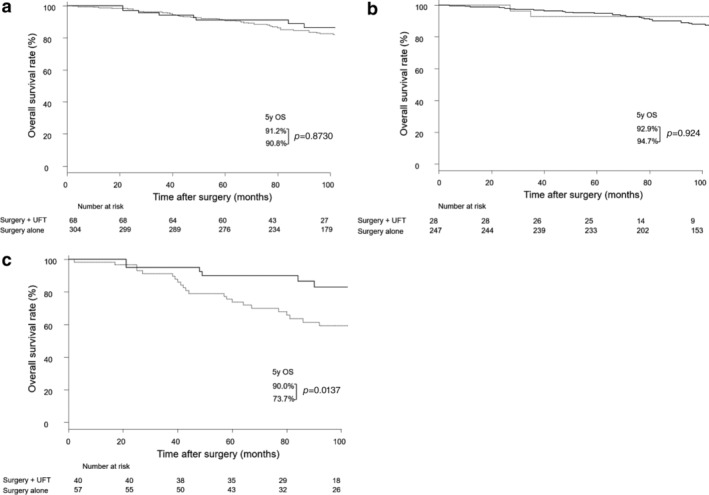
(**a**) Survival curve among patients with stage I pulmonary adenocarcinoma with low expression of Psf3 and who received adjuvant UFT (*N* = 372). (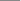) Surgery + UFT (*n* = 68) and (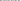) surgery alone (*n* = 304). (**b**) Survival curve among patients with stage I pulmonary adenocarcinoma with low expression of Psf3 and who received adjuvant UFT (*N* = 275). (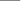) Surgery + UFT (*n* = 28) and (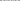) surgery alone (*n* = 247). (**c**) Survival curve among patients with stage IB pulmonary adenocarcinoma with low expression of Psf3 and who received adjuvant UFT (*N* = 97). (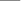) Surgery + UFT (*n* = 40) and (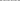) surgery alone (*n* = 57). UFT, tegafur‐uracil.

Next, we examined the association between the Psf3 expression and the efficacy of UFT. Among stage I patients in the Psf3 high expression group, the five‐year survival rate was significantly greater in patients who underwent surgery with adjuvant UFT than in those who underwent surgery alone (91.5% vs. 70.9%, *P* < 0.0001; Fig 3a); similar outcomes were observed among patients in stage IA (92.7% vs. 79.8%, *P* < 0.0503; Fig 3b) and stage IB (90.3% vs. 56.1%, *P* < 0.05; Fig 3c). In contrast, among stage I patients in the Psf3 low expression group, no difference was found in the five‐year survival between patients who underwent surgery with adjuvant UFT and those who underwent surgery alone (91.2% vs. 90.8%, respectively; *P* = 0.873; Fig 4a); a similar outcome was observed among patients in stage IA (92.9% and 94.7%, respectively; *P* = 0.924; Fig 4b). However, among Psf3 low expression patients in stage IB, the five‐year survival was significantly higher in patients who underwent surgery with adjuvant UFT than in those who underwent surgery alone (90.0% vs. 73.7%, *P* = 0.0137; Fig 4c).

## Discussion

In this study, we demonstrated that high expression of Psf3 was a poor prognostic factor among patients with stage I pulmonary adenocarcinoma. Moreover, the efficacy of UFT as adjuvant chemotherapy was shown for both stage IA and IB patients with high expression of Psf3 but not in stage IA patients with low expression of Psf3.

According to previous reports in Japan, adjuvant UFT can be used for stage I patients with tumor diameter ≥ 2 cm, but its efficacy had not been reported for stage IA patients with tumor diameter < 2 cm.^5^, ^6^ Notably, a certain number of stage IA patients with tumor diameter < 2 cm experience early postoperative recurrence. We considered that adjuvant administration of UFT could improve the prognosis of those patients. Although T stage is an important prognostic factor for early pulmonary adenocarcinoma, the expression of Psf3 was a more reliable prognostic factor in our multivariate analysis. Furthermore, adjuvant UFT for stage IA patients improved the prognosis of patients with high expression of Psf3, but it had no advantages for patients with low expression of Psf3. These results indicated that Psf3 expression might be a biomarker of UFT administration for stage IA NSCLC.

Psf3 is a member of the GINS complex, which is a heterotetrameric assembly of SLD5, Psf1, Psf2, and Psf3. In eukaryotic cells, the GINS complex had been associated with the cell division cycle (Cdc) protein 45 and the mini‐chromosome maintenance (MCM) protein to form the CMG (Cdc45–MCM–GINS) complex that constitutes the DNA replication helicase.^11^, ^12^, ^13^, ^14^ For their role in DNA replication, GINS has previously been reported as a requirement for the acute growth of cells, especially immature cells, such as stem cells and progenitor cells; moreover, this protein plays a role in cancer cells.^15^, ^16^ Recently, we investigated the role of Psf3 in lung cancer cells, with the use of a molecular biological approach. Psf3 was upregulated in the pulmonary adenocarcinoma cell line A549 and lung squamous cell carcinoma cell line EBC 1, and knockdown of Psf3 delayed the S phase of the DNA in the cell lines. These indicated that Psf3 played a role in the DNA replication of lung cancer cells and was an essential factor in lung cancer proliferation.^17^

Previous reports have demonstrated that UFT improved the prognosis of patients with active DNA synthesis in tumors.^18^, ^19^, ^20^ We considered that the efficacy of UFT might be relatively high in malignant cells in which DNA replication is active. Based on the close association of Psf3 with the activity of DNA replication and cancer proliferation, we considered that adjuvant UFT administration would be relatively more advantageous for patients with high expression of Psf3 and that Psf3 could be a biomarker of UFT as adjuvant therapy for stage IA patients. Although several biomarkers, such as alpha‐actinin‐4 and nectin‐like molecule‐5, have been shown to indicate a poor prognosis of pulmonary cancer and were correlated with cell migration, invasion, or metastasis,^21^, ^22^ Psf3 could be a more effective biomarker considering the mechanism of action of UFT. The expression of Ki67, which is a known cell proliferation marker similar to Psf3, was reported to be higher in the Psf3 high expression group than in the Psf3 low expression group.^8^, ^23^, ^24^ However, upon comparison following immunostaining, Psf3 was mainly detected in the nuclei of tumor cells, whereas ki‐67 was found in both the nuclei and stroma of the tumor cells.^23^ These results suggested that immunostaining of Psf3 was useful for selectively detecting tumor‐proliferating cells.

We recognize that there are some limitations in this study. First, this was a single‐institution study and the number of stage IA patients who received adjuvant UFT was small. From these results, we will conduct a prospective randomized multi‐institutional trial to verify the efficacy of UFT as adjuvant therapy for stage IA pulmonary cancer patients with high expression of Psf3 and tumor diameter < 2 cm. Second, we could not accurately measure the invasive diameter of the tumor because the study period was from January 2002 to December 2009. Therefore, we used the seventh edition of the TNM classification in this study. However, part‐solid lung cancer, which is expected to have a better prognosis compared with pure solid lung cancer, was considered to be classified to the Psf3 low expression group. Therefore, we considered that the effect of the difference in the TNM edition was relatively less among patients in the Psf3 high expression group. However, we will continue to collect patients’ information including expression of Psf3 in the future and assess the outcome using the eighth edition of the TNM classification because the treatment of lung cancer has changed since the study was first conducted.

In conclusion, high expression of Psf3 was a poor prognostic factor among patients with stage I pulmonary adenocarcinoma. The efficacy of UFT as adjuvant chemotherapy was shown for stage IB patients, regardless of Psf3 expression, and for stage IA patients with high expression of Psf3. Our findings indicated that Psf3 expression might be a biomarker of UFT use for patients with stage IA pulmonary adenocarcinoma.

## Disclosure

No authors report any conflict of interest.
